# Machine-Learning-Assisted Multi-Element Optimization of Mechanical Properties in Spinel Refractory Materials

**DOI:** 10.3390/ma18081719

**Published:** 2025-04-09

**Authors:** Zhiyuan Chen, Daoyuan Yang, Xianghui Li, Jinfeng Li, Huiyu Yuan, Junyan Cui

**Affiliations:** School of Materials Science and Engineering, Zhengzhou University, Zhengzhou 450001, China; chen_zy@gs.zzu.edu.cn (Z.C.); 13384002106@139.com (X.L.); 18946756320@163.com (J.L.); hyyuan@zzu.edu.cn (H.Y.); jycui@zzu.edu.cn (J.C.)

**Keywords:** spinel refractory materials, multi-element optimization, machine learning models, flexural strength, microhardness

## Abstract

Using machine learning models, this study innovatively introduces multi-element compositions to optimize the performance of spinel refractories. A total of 1120 spinel samples were fabricated at 1600 °C for 2 h, and an experimental database containing 112 data points was constructed. High-throughput performance predictions and experimental verifications were conducted, identifying the sample with the highest hardness, (Al_2_Fe_0.25_Zn_0.25_Mg_0.25_Mn_0.25_)O_4_ (1770.6 ± 79.1 HV1, 3.35 times that of MgAl_2_O_4_), and the highest flexural strength, (Al_2_Cr_0.5_Zn_0.1_Mg_0.2_Mn_0.2_)O_4_ (161.2 ± 9.7 MPa, 1.4 times that of MgAl_2_O_4_). Further analysis of phase composition and microstructure shows that the mechanism of hardness enhancement is mainly the solid solution strengthening of multi-element doping, the energy dissipation of the large-grain layered structure, and the reinforcement of the zigzag grain boundary. In addition to solid solution strengthening and a compact low-pore structure, the mechanism of improving bending strength also includes second-phase strengthening and phase concentration gradient distribution. This method provides a promising way to optimize the performance of refractory materials.

## 1. Introduction

Spinel refractory materials, represented by magnesium aluminum spinel (MgAl_2_O_4_), are critical high-temperature structural materials typically synthesized through high-temperature solid-state reactions or electric fusion methods to form stable spinel-structured products [1]. Their high melting point, excellent thermal shock resistance, chemical stability, and mechanical strength enable long-term stability in high-temperature environments while resisting temperature fluctuations, chemical corrosion, and mechanical impacts [2,3]. Due to these exceptional properties, spinel materials are widely applied in various high-temperature industrial fields, such as steelmaking, cement production, and glass manufacturing, where they are used in furnaces, kilns, and reactors. Whether as aggregates [4,5] or binding phases [6,7], spinel refractories play a crucial role in enhancing the performance and lifespan of refractory products, making them indispensable for maintaining structural integrity in demanding industrial processes.

High-entropy ceramics (HECs) are solid solutions characterized by high configurational entropy, typically composed of five or more cationic or anionic sublattices [8]. Lattice distortion, solution strengthening, and other effects make high entropy ceramics exhibit excellent properties, such as ultra-high melting point, high mechanical strength, and strong corrosion resistance, so it has attracted wide attention. To date, various high-entropy ceramics have been synthesized, including high-entropy oxides [9,10,11], nitrides [12,13], carbides [14,15], borides [16], silicides [17], and fluorides [18]. Among them, high-entropy oxides (HEOs) exhibit increased local entropy, which makes the anionic sublattices particularly susceptible to local deformation and defects. In localized regions, each ion tends to isotropically distort its coordination polyhedron, deviating from the mean [19]. The unique electronic instability of cations also contributes to anisotropic local distortions in HEOs [20]. Besides the simple expansion, contraction, or distortion of the local oxygen sublattice, point defects play a crucial role in influencing the high-entropy properties [21]. These defects include vacancies on the cationic or oxygen sublattices, interstitial elements, impurities or dopants, and antisite defects [22].

In addition, some studies have extended the multi-element doping effect of high-entropy ceramics to compositionally complex ceramics (CCCs) or multi-principal cation ceramics (MPCCs) to explore medium entropy and non-equimolar compositions [23], and existing studies have also shown good mechanical or thermophysical properties of medium-entropy ceramics [24,25]. Multi-element doping is also a hot spot in refractory research, among which the spinel structure (the AB_2_O_4_ phase, where A represents the divalent cation and B represents the trivalent cation), which contains various voids and vacancies, can form a solid solution with a large solubility range, so it is widely used in multi-element doping work, especially in the environmentally friendly design of refractory without chromium [26,27].

In summary, the application of high-entropy and medium-entropy multi-element doping to optimize the composition of spinel refractory materials is a promising approach for improving their performance. By introducing complex elements, the properties of spinel aggregates can be improved or compositional guidance for the performance optimization of spinel phases generated in refractories can be provided. Flexural strength and hardness are critical indicators for evaluating the quality and applicability of refractory materials: flexural strength reflects the material’s ability to resist bending stress and fracture at room temperature, while hardness indicates its resistance to surface deformation and wear. Therefore, the primary focus of this study is to determine whether the multi-element doped complex composition system can effectively enhance both the flexural strength and hardness of traditional spinel refractories. However, finding effective and feasible experimental methods to explore the vast composition space of complex multi-element systems, including high-entropy and medium-entropy compositions, remains a significant challenge.

With the advancement of computational power, machine learning (ML) technology has increasingly intersected with materials science. Using experimental data or simulation data as a database, ML models can be trained to simulate the relationship between descriptors and target properties, thereby significantly reducing the experimental exploration space for these descriptors and properties. This has proven to be a feasible experimental method. Different ML models, however, utilize varying descriptors. Some models use large datasets from first-principle calculations for more accurate predictions. For instance, Zhao et al. [28] studied the formation energy of 1280 carbon vacancies using high-throughput density functional theory (HT-DFT) calculations and ML models. They trained a Random Forest model with DFT results to predict vacancy formation energies, achieving a mean absolute error of 54 meV. Zhou et al. [29] introduced a machine learning (ML) design approach powered by a data augmentation generative adversarial network (DAGAN) to predict the hardness, Young’s modulus, and wear resistance of innovative high-entropy nitride ceramics. After evaluating multiple feature selection algorithms, they identified an optimal set of descriptors that included data derived from density functional theory (DFT) calculations. To address the limited data availability in high-entropy nitrides (HENs), they applied the DAGAN algorithm to generate an augmented dataset, thereby improving the accuracy of their ML model by up to 14.67%. Through this method, they successfully identified eight superhard HEN systems with hardness values exceeding 40 GPa. Hong Meng et al. [30] combined high-throughput computation with ML to establish effective descriptors for the formation ability of high-entropy boride ceramics. They utilized 22 formation ability descriptors derived from both DFT calculations and experimental data for ML training.

Previous researchers have also utilized training databases formed by weighted combinations of elemental properties or precursor physicochemical properties, enabling high-throughput predictions. Although the accuracy may be lower compared to computational databases, these methods have the advantage of easily obtainable descriptors and do not involve DFT calculations, allowing for rapid screening of the vast search space of descriptors and target properties [31,32,33]. Li et al. [34] proposed an ML method to predict the saturation magnetization (Bs) and critical diameter (Dmax) of soft magnetic metallic glasses (mg). They established two datasets based on published soft magnetic experimental results and introduced a universally accessible feature space, demonstrating its adaptability to different ML model training tasks. Yan et al. [35] trained their models using data collected from high-throughput experiments. Their K-nearest neighbors (KNNs) model achieved an experimental validation accuracy of 93.75%, proving that the bond mismatch between boron and transition metals (δB-TM) predominantly governs the formation of high-entropy borides (HEBs).

Given that multi-element optimization remains a relatively unexplored area in the field of refractory materials, we propose a machine-learning-based high-throughput predictive approach aimed at enabling large-scale screening and selection, providing an efficient tool for optimizing the performance of spinel refractory materials. In this study, we selected readily accessible elemental and precursor properties as descriptors to explore the multi-element optimization of spinel refractory materials. Using an experimental database comprising 112 datasets, we trained eight different machine learning algorithms and identified the most effective models for high-throughput screening of flexural strength and hardness across various compositions. Subsequently, samples exhibiting superior flexural strength and hardness were subjected to experimental characterization and analysis.

## 2. Materials and Methods

### 2.1. Sample Preparation and Basic Performance Test Methods

**Figure A1:**
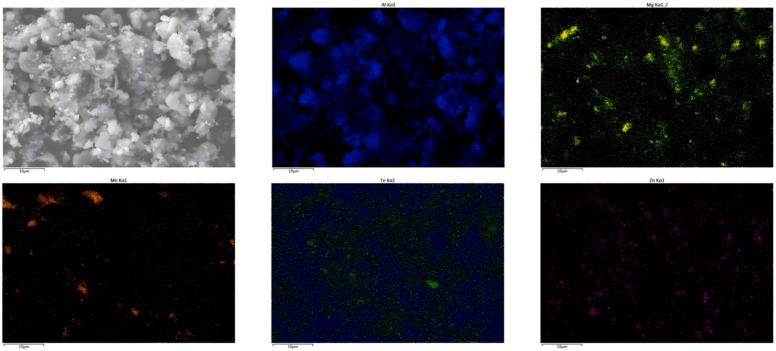
SEM and EDS images of high-hardness non-sintered (Al_2_Fe_0.25_Zn_0.25_Mg_0.25_Mn_0.25_)O_4_ powder samples after ball milling and drying.

**Figure A2:**
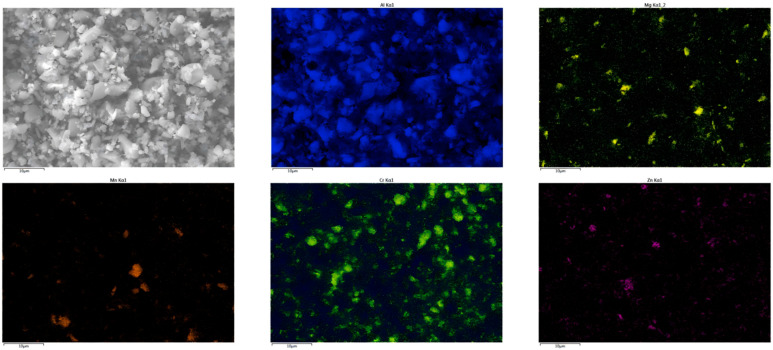
SEM and EDS images of high flexural strength non-sintered (Al_2_Cr_0.5_Zn_0.1_Mg_0.2_Mn_0.2_)O_4_ powder samples after ball milling and drying.

**Figure A3:**
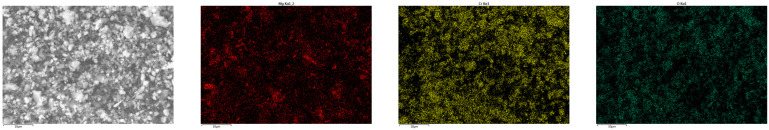
SEM and EDS images of non-sintered MgCr_2_O_4_ powder samples after ball milling and drying.

**Figure A4:**
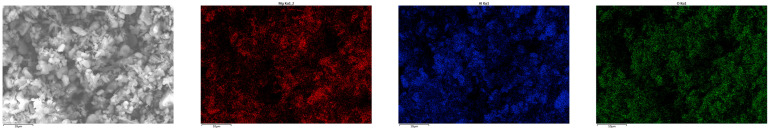
SEM and EDS images of non-sintered MgAl_2_O_4_ powder samples after ball milling and drying.

The oxide raw materials used in the experiments were sourced from China Shanghai Macklin Biochemical Technology Co., Ltd., with the following specifications: Al_2_O_3_ (AR 99%), Fe_2_O_3_ (AR 99%), Cr_2_O_3_ (AR 99%), MgO (AR 99%), ZnO (AR 99%), CuO (AR 99%), and MnO_2_ (85%). The oxide reagents were prepared according to the designed metal element ratios specified in the database, producing a total of 150 g of powder. This powder was mixed with anhydrous ethanol at a 1:1 volume ratio and subjected to planetary ball milling for 6 h. After ball milling, the mixture was dried in a 100 °C oven for 12 h. The morphology and elemental distribution of the dried, non-sintered ball-milled powder are shown in Appendix A, Figure A1, Figure A2, Figure A3 and Figure A4. After drying, the dried ball-milled powder was pressed into samples with dimensions of 40 mm × 5 mm × 5 mm. No binder was added during the pressing process, and dry pressing was performed using the ball-milled powder. The pressure was gradually increased in four stages: 50 MPa, 100 MPa, 150 MPa, and 200 MPa, with each stage maintained for 6 s. The final pressure of 200 MPa was held for 6 s before releasing the sample. A total of 40 non-sintered samples were prepared for each formulation.

**Figure 1:**
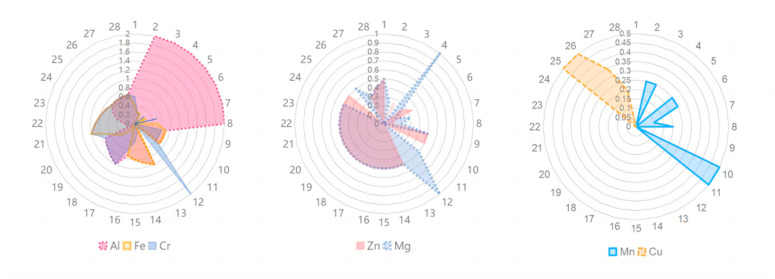
Element content radar map of the database.

Subsequently, the non-sintered samples were sintered at four different temperatures: 1000 °C, 1200 °C, 1400 °C, and 1600 °C, with 10 samples sintered at each temperature. During the sintering process, the temperature gradient was as follows: heating at a rate of 3 °C per minute below 300 °C and 5 °C per minute above 300 °C. Once the preset temperature was reached, the samples were held at that temperature for 2 h and then naturally cooled. Consequently, this study prepared 112 sets of multi-element spinel ceramics with varying compositions and sintering temperatures, totaling 1120 samples. Each composition and sintering temperature combination had 10 samples available for property testing. To construct the database, we prioritized aluminum, iron, chromium, zinc, magnesium, manganese, and copper—elements widely used in the refractory industry and relatively low in cost. In designing the formulations for the samples in the database, the A-site and B-site metal elements in the spinel structure were distinguished. Two approaches were employed to design the sample compositions: first, by fixing the A-site metal content while varying the types and ratios of the B-site metals; and second, by fixing the content and types of the B-site metals while altering the types and ratios of the A-site metals. These approaches facilitated the construction of a database encompassing diverse formulations. As shown in Figure 1, the radar charts illustrate the different compositions, with each formulation number arranged along the perimeter and each concentric circle representing the molar ratio of the corresponding metal element in the sample formulations.

The flexural strength of the samples at room temperature was measured according to the ISO 14704:2016(E) [36] three-point bending method, with a span of 30 mm between the support points. Microhardness was assessed following the ISO 6507-1 standard [37], applying a 9.8 N load and holding for 10 s. The Vickers hardness testing method was employed to calculate the hardness values from the diagonal lengths of the indentations. The apparent porosity of the samples was determined using the water displacement method according to the ISO 5017:2013 standard [38]. The X-ray diffraction (XRD) data were collected using a PANalytical Empyrean diffractometer(Malvern Panalytical B.V., Almelo, The Netherlands) equipped with a Cu target. The raw XRD data, used for refinement, were obtained from flat bulk samples within a 2θ range of 10–80°, with a step size of 0.013° and a step time of 10.200 s. XRD refinement was performed using the Highscore Plus (ver 4.8) software. Scanning Electron Microscopy (SEM) and Energy Dispersive Spectroscopy (EDS) analyses were conducted using Thermo Fisher Scientific’s Helios G4 CX DualBeam workstation(Thermo Fisher Scientific Inc., Waltham, MA, USA). The samples were coated with platinum as a conductive layer, with a working distance of 3–5 mm. The SEM was operated at an accelerating voltage of 5 kV, while the EDS was performed at an accelerating voltage of 20 kV.

### 2.2. Machine Learning Methods

#### 2.2.1. Database Construction

**Figure 2:**
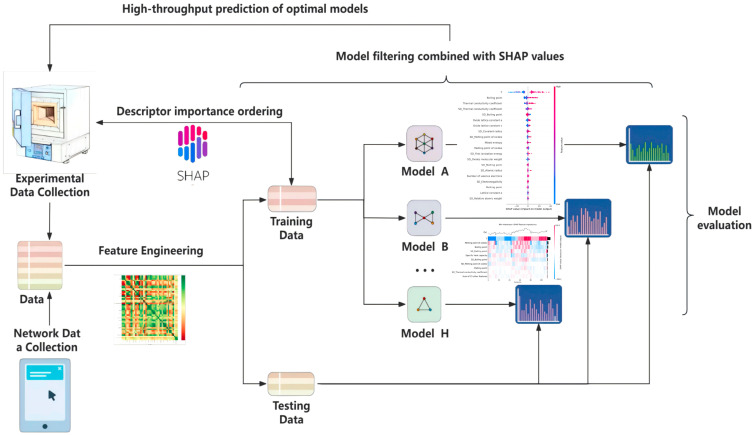
Complete machine learning workflow.

The complete machine learning process is illustrated in Figure 2. The database comprises 48 selected descriptors. The first 46 descriptors are derived from the intrinsic properties of metal atoms and the physicochemical properties of the precursor oxides used. The 47th descriptor in the database is the thermodynamic configurational entropy ΔS_conf_ (Equation (1)). The 48th descriptor is the sintering temperature of the samples after pressing, which is a key process parameter affecting material properties. These descriptors not only provide fundamental information about the material properties but also reveal the composite effects of the material components through the calculation of weighted averages (Equation (2)) and weighted standard deviations (Equation (3)). The full list of 48 descriptors can be found in Table A1. Elemental and atomic data were obtained from three recognized free databases: Materials Project, AFLOW, and the Royal Society of Chemistry.

The thermodynamic configurational entropy ΔS_conf_ [39,40] is calculated by the following formula (Equation (1)):

(1) $${\Delta S}_{\mathrm{conf}}=-R\sum_{i}^{n}a_{i}\ln a_{i}$$

where *R* is the gas constant, equal to 8.314 J/(mol·K); *a_i_* is the mole fraction; and *n* is the number of components.

The calculation formula for the weighted average is as follows (Equation (2)):

(2) $${\bar{x}}_{w}=\frac{\sum_{i=1}^{j}w_{i}x_{i}}{\sum_{i=1}^{j}w_{i}}$$

The calculation formula for the weighted standard deviation is as follows (Equation (3)):

(3) $$s_{w}=\sqrt{\frac{\sum_{i=1}^{j}w_{i}\cdot {\left(x_{i}-{\bar{x}}_{w}\right)}^{2}}{\sum_{i=1}^{j}w_{i}}}$$

where *x_i_* represents the *i*-th value, *w_i_* represents the corresponding weight, and *j* is the total number of calculated data required.

With these easily obtainable descriptors, the foundation for high-throughput predictions is established. However, before applying machine learning models in practice, it is essential to filter these descriptors to ensure the model’s effectiveness and accuracy.

#### 2.2.2. Descriptor Selection

The Pearson correlation coefficient was used to calculate the degree of association between each descriptor. This coefficient is a statistical measure that evaluates the linear correlation between two variables, with values ranging between −1 and +1. A positive value indicates a positive correlation, a negative value indicates a negative correlation, and a value of zero indicates no linear correlation [41].

The calculation formula for the Pearson correlation coefficient is as follows (Equation (4)):

(4) $$r=\frac{\sum_{i=1}^{k}\left(b_{i}-\bar{b}\right)\left(c_{i}-\bar{c}\right)}{\sqrt{\sum_{i=1}^{k}{\left(b_{i}-\bar{b}\right)}^{2}\sum_{i=1}^{k}{\left(c_{i}-\bar{c}\right)}^{2}}}$$

where *b_i_* and *c_i_* are the two variables, $\bar{b}$ is the average of the variable *b*, and $\bar{c}$ is the average of the variable *c*. *k* is the total number of samples in the database.

#### 2.2.3. Model Evaluation

During hyperparameter tuning, we applied ten-fold cross-validation, using the Coefficient of Determination (R^2^) and Root Mean Square Error (RMSE) to assess the model’s prediction accuracy.

The calculation formula for the R^2^ value is as follows (Equation (5)):

(5) $$R^{2}=1-\frac{\sum_{i=1}^{k}{\left(d_{i}-{\hat{d}}_{i}\right)}^{2}}{\sum_{i=1}^{k}{\left(d_{i}-{\bar{d}}_{i}\right)}^{2}}$$

The calculation formula for the RMSE value is as follows (Equation (6)):

(6) $$\mathrm{RMSE}=\sqrt{\frac{1}{k}\sum_{i=1}^{k}{\left(d_{i}-{\hat{d}}_{i}\right)}^{2}}$$

where *d_i_* is the actual observed value for the *i*-th sample, ${\hat{d}}_{i}$ is the predicted value for the *i*-th sample based on the model, and ${\bar{d}}_{i}$ is the mean of the actual observed values.

## 3. Results

### 3.1. Experimental Results

**Figure 3:**
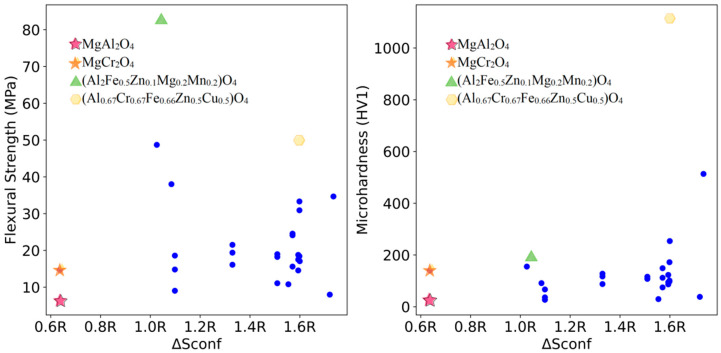
Mixing entropy and mechanical properties trend at 1200 °C for samples.

**Figure 4:**
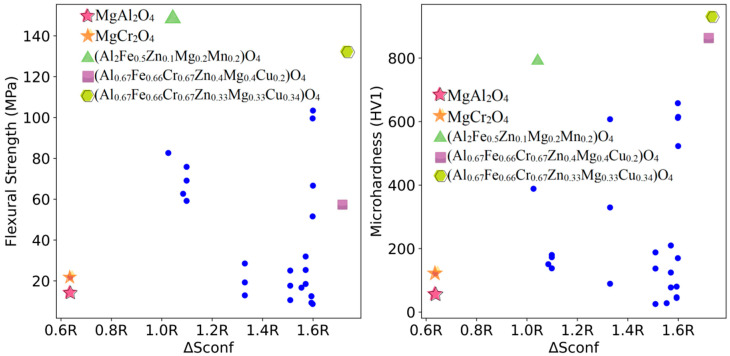
Mixing entropy and mechanical properties trend at 1400 °C for samples.

**Figure 5:**
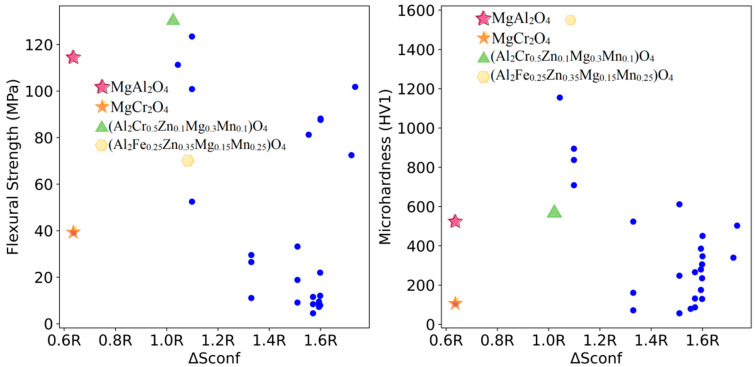
Mixing entropy and mechanical properties trend at 1600 °C for samples.

The relationships between the mixing entropy of each formulation and the mechanical properties of the sintered samples at 1200 °C, 1400 °C, and 1600 °C were deduced from the 112 experimental sets and are shown in Figure 3, Figure 4 and Figure 5, respectively.

As shown in Figure 3, MgAl_2_O_4_ samples with lower mixing entropy have lower flexural strength and Vickers hardness under sintering conditions at 1200 °C. MgCr_2_O_4_ also has a low mixing entropy, with flexural strength slightly higher than that of MgAl_2_O_4_ but still not significant. All multi-element samples demonstrate higher flexural strength compared to MgAl_2_O_4_, and most of these multi-element samples also surpass MgCr_2_O_4_ in flexural strength. Notably, (Al_2_Fe_0.5_Zn_0.1_Mg_0.2_Mn_0.2_)O_4_ and (Al_0.67_Cr_0.67_Fe_0.66_Zn_0.5_Cu_0.5_)O_4_ exhibit flexural strengths that are 13.17 times higher and 7.94 times higher than that of the MgAl_2_O_4_ refractory materials at the same sintering temperature, respectively. Their microhardness values are 8.62 times and 48.79 times that of MgAl_2_O_4_, respectively. Compared to MgCr_2_O_4_ refractory materials, their flexural strengths are 5.63 times and 3.39 times higher, with microhardness values being 1.4 times and 7.95 times higher, respectively. Detailed data are provided in Table 1.

As shown in Figure 4, under the sintering condition of 1400 °C, MgAl_2_O_4_ and MgCr_2_O_4_, which have lower mixing entropy, also exhibit lower flexural strength and Vickers hardness. The performance of multi-element samples is superior as well. Notably, (Al_2_Fe_0.5_Zn_0.1_Mg_0.2_Mn_0.2_)O_4_ and (Al_0.67_Fe_0.66_Cr_0.67_Zn_0.33_Mg_0.33_Cu_0.34_)O_4_ show flexural strengths that are 10.41 times and 9.25 times higher than those of MgAl_2_O_4_ at the same sintering temperature, respectively. Their Vickers hardness values are 13.61 times and 15.91 times higher, respectively. Compared to MgCr_2_O_4_ refractory materials, their flexural strengths are 6.79 times and 6.04 times higher, with the Vickers hardness values being 6.46 times and 7.55 times higher, respectively. Additionally, (Al_0.67_Fe_0.66_Cr_0.67_Zn_0.4_Mg_0.4_Cu_0.2_)O_4_ exhibits very high Vickers hardness, being 14.86 times and 7.05 times higher than those of MgAl_2_O_4_ and MgCr_2_O_4_ at the same sintering temperature, respectively. Detailed data are provided in Table 2.

As shown in Figure 5, under the sintering condition of 1600 °C, the flexural strength and microhardness of the low-mixing-entropy MgAl_2_O_4_ and MgCr_2_O_4_ refractory materials significantly improve compared to their lower temperature sintering conditions. However, at the sintering temperature of 1600 °C, (Al_2_Cr_0.5_Zn_0.1_Mg_0.3_Mn_0.1_)O_4_ and (Al_2_Fe_0.25_Zn_0.35_Mg_0.15_Mn_0.25_)O_4_ exhibit flexural strengths that are 1.13 times and 0.61 times that of the MgAl_2_O_4_ refractory materials sintered at the same temperature, respectively. Their Vickers hardness values are 1.07 times and 2.92 times higher, respectively. Compared to MgCr_2_O_4_ refractory materials, their flexural strengths are 3.32 times and 1.8 times higher, with the Vickers hardness values being 5.37 times and 14.65 times higher, respectively. Detailed data are provided in Table 3.

### 3.2. Machine Learning Process

Throughout the entire process, the Pearson correlation coefficients for the original database containing 48 descriptors were first calculated, and 24 descriptors with Pearson correlation coefficients less than 0.9 were preliminarily selected. We then independently ranked the SHAP importance of these 24 descriptors using eight algorithms: Adaptive Boosting (Ada), Decision Tree Regression (DTR), Elastic Net (EN), Least Absolute Shrinkage and Selection Operator (LASSO), Neural Network (NN), Random Forest (RF), Ridge Regression (Ridge), and Extreme Gradient Boosting (XGB). For each algorithm, we selected the top 4, 8, 12, 16, 20, and 24 descriptors based on their SHAP importance rankings, forming six temporary databases for training and selection.

Finally, for the training of flexural strength, the Random Forest algorithm combined with the database containing its top eight SHAP-ranked descriptors achieved the highest R^2^ value and relatively low RMSE value. For microhardness training, the Neural Network algorithm combined with its top eight SHAP-ranked descriptors yielded the highest R^2^ value and a relatively low RMSE value. Subsequent hyperparameter tuning on these two optimal algorithm–database combinations resulted in the final machine learning models used for precise prediction. The details of the entire process are outlined as follows.

#### 3.2.1. Selected Descriptors

From the original 48 descriptors, those with an absolute Pearson coefficient greater than 0.9 were removed to retain features with more independent information [42]. The remaining 24 basic descriptors are listed in Table 4.

#### 3.2.2. Selected Algorithms

**Figure 6:**
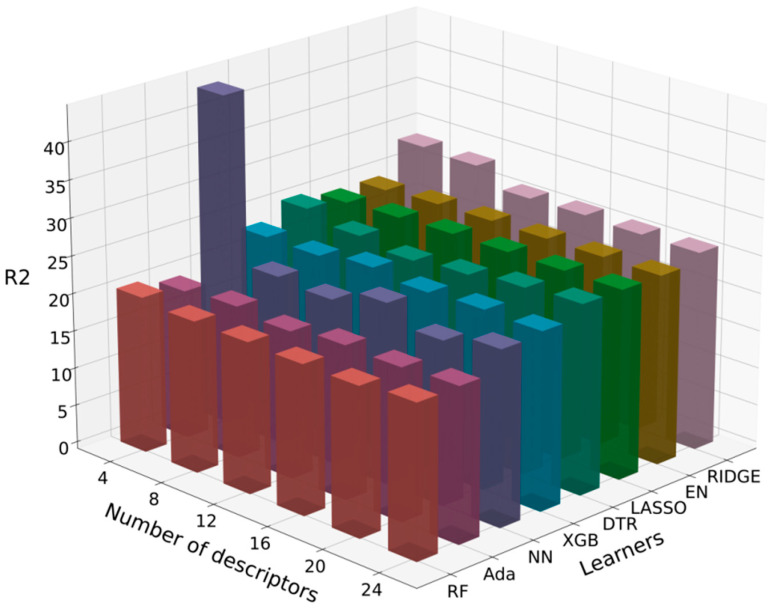
Three-dimensional bar chart of RMSE values for flexural strength model and descriptor training.

**Figure 7:**
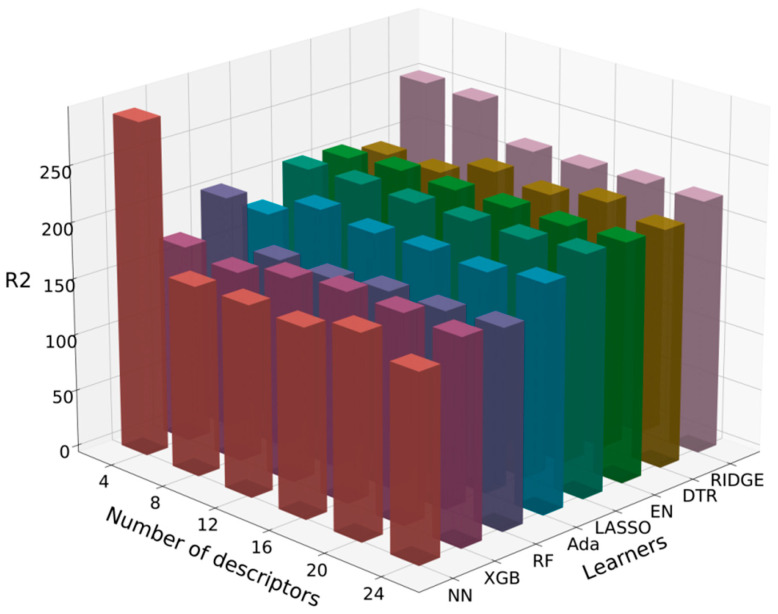
Three-dimensional bar chart of RMSE values for microhardness model and descriptor training.

After obtaining the 24 less correlated descriptors, we used the Shapley Additive Explanations (SHAP) package to select combinations of eight algorithms with databases containing different numbers of descriptors. The training R^2^ values are shown in Table 5 and Table 6, and the RMSE values are shown in Figure 6 and Figure 7. The SHAP package is a Python 3.11.5 library that interprets model predictions based on Shapley values. Shapley values, originating from game theory, quantify each feature’s contribution to the model’s prediction [43]. It provides a method to calculate the importance of each descriptor in the prediction results, reflecting the contribution of each feature to the model’s prediction [44].

For the training of flexural strength, the Random Forest (RF) algorithm combined with the database containing its top eight SHAP-ranked descriptors achieved the highest R^2^ value of 0.571, as shown in Table 5, and a relatively low RMSE value of 17.7 MPa, as shown in Figure 6. Therefore, the Random Forest (RF) algorithm was selected for the next step of training the flexural strength prediction model.

For the training of microhardness, the Neural Network (NN) algorithm combined with the database containing its top eight SHAP-ranked descriptors achieved the highest R^2^ value of 0.574, as shown in Table 6, and a relatively low RMSE value of 136.8 HV1, as shown in Figure 7. Therefore, the Neural Network (NN) algorithm was selected for the next step of training the microhardness prediction model.

#### 3.2.3. High-Performance Predictive Models

For the high R^2^ and low RMSE values achieved by the artificial Neural Network and Random Forest algorithms, we performed hyperparameter optimization using Grid Search. We employed ten-fold cross-validation R^2^ as the evaluation standard, defining a large hyperparameter search space and conducting over ten million iterations. The final Random Forest model, trained on the 112-sample flexural strength database, achieved an R^2^ value of 0.9326 and an RMSE value of 8.9 MPa. The Neural Network model, trained on the 112-sample microhardness database, achieved an R^2^ value of 0.8516 and an RMSE value of 111.8 HV1. The smaller the RMSE, the better the fitting effect of the model, but if the RMSE is too small, it may indicate that the model has an overfitting problem. Both overfitting and underfitting are not ideal model states. Therefore, it can be considered that the fitting result of this model is relatively ideal. The specific hyperparameters selected for both models are detailed in Table 7.

### 3.3. High-Throughput Prediction of Optimal Mechanical Properties Compositions

**Figure 8:**
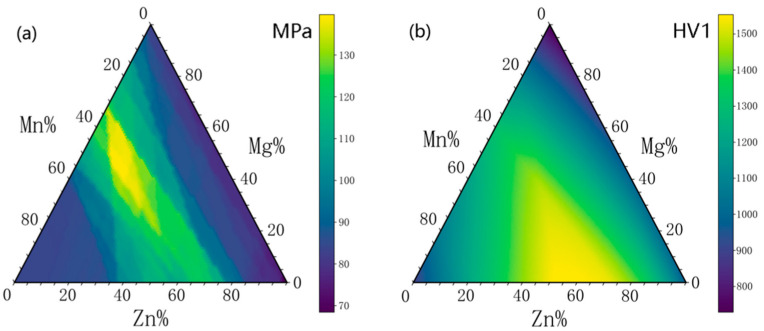
Ternary heat map for predicting properties at 1600 °C: (**a**) flexural strength of (Al_2_Cr_0.5_Zn_x_Mg_y_Mn_z_)O_4_; (**b**) microhardness of (Al_2_Fe_0.25_Zn_x_Mg_y_Mn_z_)O_4_.

After obtaining two high-performance models, we explored the flexural strength and microhardness of spinel refractory sintered at 1600 °C with five-element compositions. The Random Forest model for predicting flexural strength indicated that multi-element spinel (Al_2_Cr_0.5_Zn_x_Mg_y_Mn_z_)O_4_ sintered at 1600 °C could achieve a room temperature flexural strength exceeding 130 MPa. This is over 50% higher than that of MgAl_2_O_4_ and MgCr_2_O_4_ spinels prepared under the same experimental conditions. The predicted ternary heatmap is shown in Figure 8a.

The Neural Network model for predicting microhardness indicated that multi-element spinel (Al_2_Fe_0.25_Zn_x_Mg_y_Mn_z_)O_4_ sintered at 1600 °C could achieve a microhardness exceeding 1500 HV1. This is over 300% higher than that of MgAl_2_O_4_ and MgCr_2_O_4_ spinels prepared under the same experimental conditions. The predicted ternary heatmap is shown in Figure 8b.

### 3.4. Experimental Verification of Prediction Results

**Figure A5:**
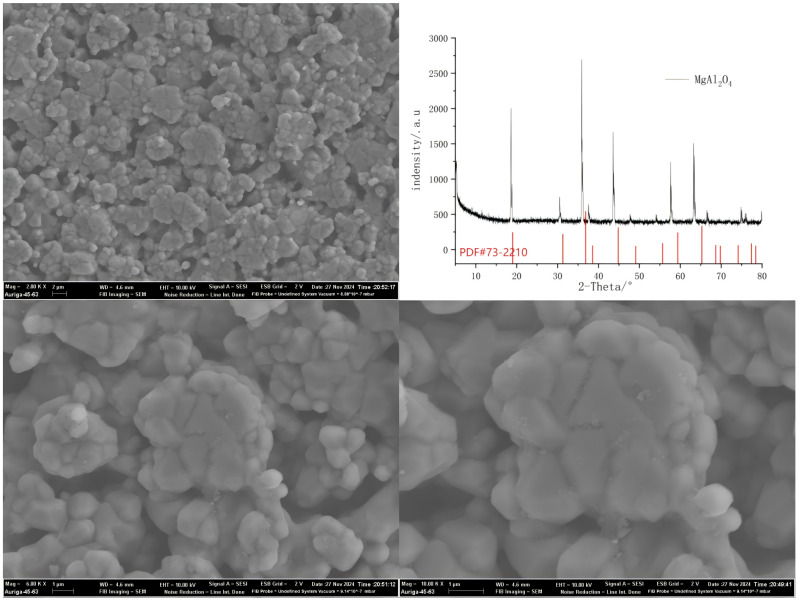
SEM micrographs and XRD data of MgAl_2_O_4_ prepared under the same experimental conditions (the apparent porosity of the sample was 18.09 ± 0.18%).

**Figure A6:**
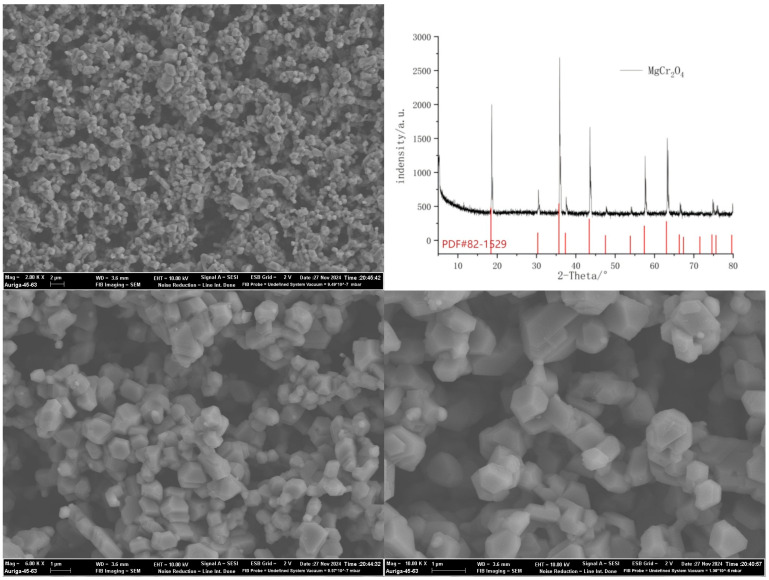
SEM micrographs and XRD data of MgCr_2_O_4_ prepared under the same experimental conditions (the apparent porosity of the sample was 33.5 ± 0.74%).

Following the predicted elemental compositions and sintering temperatures, samples were prepared using the same experimental process. Room temperature flexural strength and microhardness tests were conducted on five samples for each mechanical property, and the average values were calculated. The multi-element spinel refractory material (Al_2_Cr_0.5_Zn_0.1_Mg_0.2_Mn_0.2_)O_4_ sintered at 1600 °C exhibited a flexural strength of 161.2 MPa, with a standard deviation of 9.7 MPa, which is 1.4 times that of MgAl_2_O_4_ (SEM micrographs and XRD data shown in Appendix A Figure A5) and 4.11 times that of MgCr_2_O_4_ (SEM micrographs and XRD data shown in Appendix A Figure A6). The flexural strength of this multi-element spinel refractory is significantly improved compared to the industrial magnesite-spinel refractories, which typically have a flexural strength of 50–60 MPa. For the multi-element spinel refractory material (Al_2_Fe_0.25_Zn_0.25_Mg_0.25_Mn_0.25_)O_4_ sintered at 1600 °C, the microhardness reached 1770.6 HV1, with a standard deviation of 79.1 HV1, which is 3.35 times that of MgAl_2_O_4_ and 16.77 times that of MgCr_2_O_4_ prepared under the same conditions. The Vickers hardness is significantly higher than that of the industrial fused magnesia-alumina spinel aggregate, which typically ranges between 1100 and 1200 HV1.

The experimental verification of mechanical properties closely matched the predicted values (130 MPa for flexural strength and 1500 HV1 for microhardness), demonstrating the feasibility of using the Random Forest model for high-throughput prediction of room temperature flexural strength and the Neural Network model for predicting microhardness in this study.

## 4. Mechanism of Flexural Strength or Hardness Improvement

### 4.1. Mechanism of Hardness Improvement in Samples (Al_2_Fe_0.25_Zn_0.25_Mg_0.25_Mn_0.25_)O_4_

**Figure 9:**
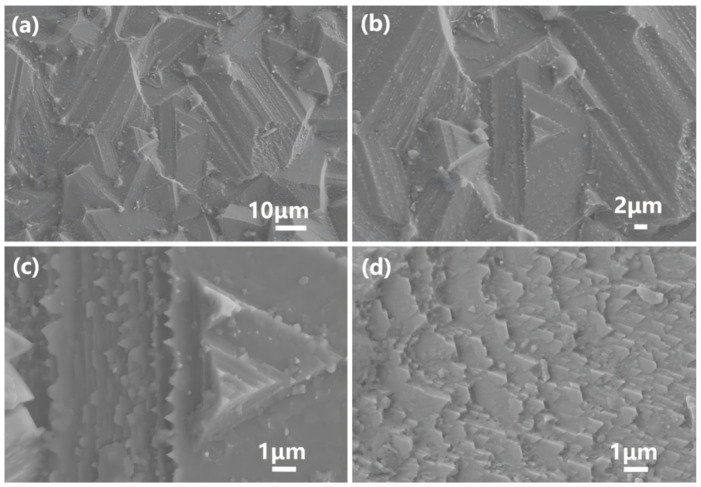
Surface SEM micrograph of the high-hardness sample (**a**,**b**); SEM micrographs of grain lamellar edge protrusions of high hardness samples (**c**,**d**).

**Figure 10:**
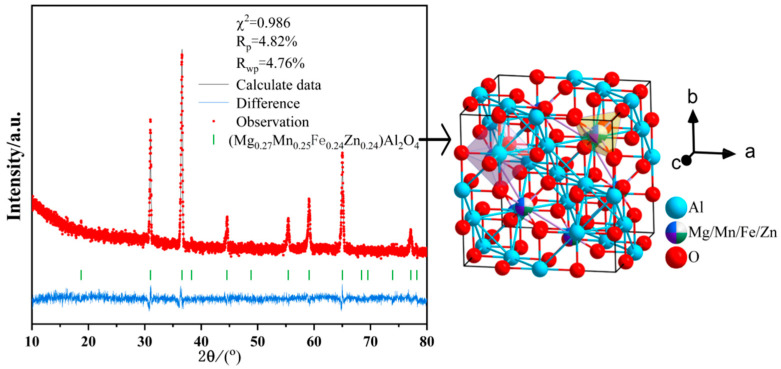
Rietveld-Refined XRD data and crystal structure diagram of the high-hardness sample.

The high-hardness sample exhibits large, densely packed grains of 30–50 μm without any observable porosity. The grains are oriented differently and intersect with each other, as shown in Figure 9a,b. Upon magnification, a layered structure can be observed on the surface of the large grains, with disordered triangular protrusions (0.1–1 μm) occurring at the edges of each layer, as depicted in Figure 9c,d. The Rietveld refinement data from X-ray diffraction (XRD), as shown in Figure 10 and Table A2, indicate that the lattice parameters are a = b = c = 8.082 Å, with a unit cell volume of 527.8 Å^3^. The occupancy rates of the doped elements (Mg, Mn, Fe, and Zn) at the tetrahedral interstitial sites were consistent with the initial stoichiometric ratio (Mg:Mn:Fe:Zn = 1:1:1:1). This confirms that these elements are successfully incorporated into the tetrahedral sites of the spinel structure, forming a substitutional solid solution [45]. Compared with the standard spinel structure data of PDF#77-0435 (MgAl_2_O_4_, lattice parameter a = 8.081Å, cell volume = 527.6Å^3^), the lattice parameter of the doped material is increased by 0.001 Å and the cell volume is increased by 0.2 Å^3^.

The mechanism for hardness enhancement is analyzed progressively from four dimensions: dopant ions, spiral growth mode, layered structure, and grains.

#### 4.1.1. Solid Solution Strengthening Mechanism of Dopant Elements

The ionic radii of doped elements occupying tetrahedral interstitial sites are different (Mg^2+^ = 0.57 A, Mn^2+^ = 0.66 A, Fe^2+^ = 0.63 A, and Zn^2+^ = 0.60 A), which causes lattice distortion, resulting in changes in lattice parameters and cell volume [46,47]. This deformation creates a local stress field within the lattice. The stress field or potential well generated by the solute atoms interacts with the dislocation to produce a pinning effect that inhibits the dislocation motion. This results in a significant increase in the hardness of the material, showing a solid solution strengthening effect.

#### 4.1.2. Energy Dissipation and Crack-Resistance Mechanism of the Spiral-Grown Layered Structure

In the process of crystal growth, the local stress field caused by doping is the driving force of dislocation, resulting in an increase in dislocation. Spiral dislocation is a step nucleus that promotes spiral growth and leads to the spiral growth mechanism. During the growth of spinel crystals, surface energy minimization drives preferential growth along the {111} crystal planes, resulting in an octahedral structure dominated by {111} planes [48,49]. A single (111) plane forms an equilateral triangle in three-dimensional space, and a complete octahedral structure consists of eight equilateral triangular crystal faces [50]. This geometric property ensures that during spiral growth, each terrace expands outward along the triangular boundaries, ultimately manifesting as a macroscale spiral-layered triangular structure (as shown in Figure 9b,c).

The spiral-layered structure optimizes internal stress distribution within the crystal, thereby enhancing its overall hardness. The interfaces between layers act as stress buffers, dispersing stress under external forces and delaying crack propagation. The layered structure can absorb and dissipate more energy through mixed deformation such as extrusion, kinking, folding, interlayer separation, and tearing [51]. Additionally, the layered structure can cause cracks to deflect at the interfaces, increasing the crack propagation path and the energy required for further growth [52]. This energy dissipation mechanism significantly contributes to the hardness improvement. Furthermore, the close packing along the {111} planes provides higher compressive resistance and deformation resistance, which also positively impacts the enhancement of the hardness.

#### 4.1.3. Strengthening Mechanism Induced by the Complexity of Layered Edge Interfaces

**Figure 11:**
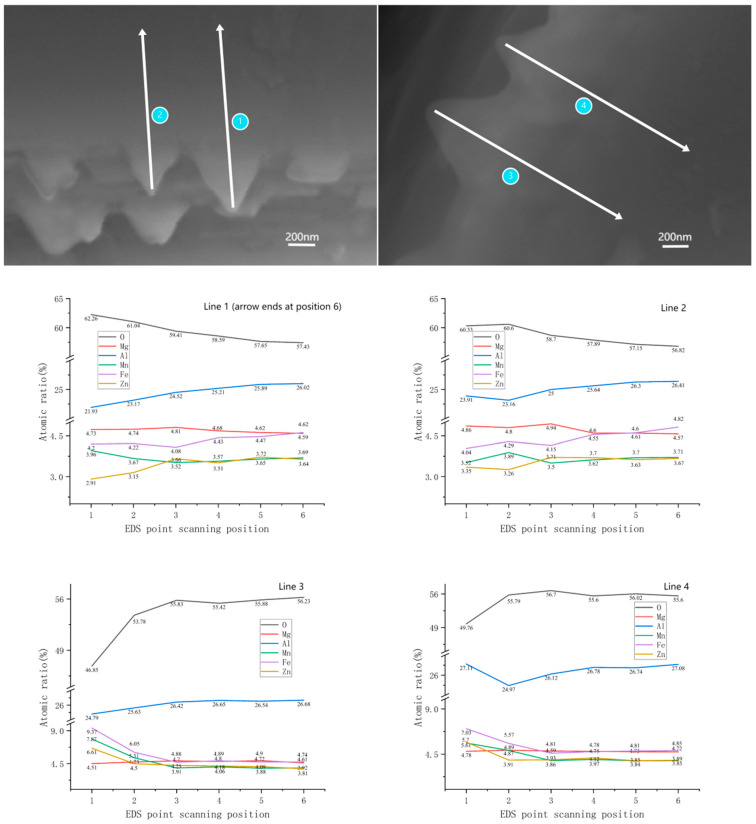
EDS mapping of the triangular morphology at the edges of the layered structure in the high-hardness sample.(The Y-axis in the line plot represents the atomic ratio of the elements at each point scan. The X-axis represents the point scan position from 1–6, where position 1 is the beginning of the arrow in the microscopic image and position 6 is the end of the arrow).

As shown in the EDS element mapping in Figure 11, differences in the diffusion rates of various dopant elements cause localized compositional fluctuations at the crystal growth interface. These fluctuations manifest as uneven growth rates at the edges of spiral terraces. The introduction of dopant elements further influences the specific characteristics of spiral growth, resulting in irregular small triangular protrusions, as observed in the SEM images in Figure 9c,d. On the one hand, their presence greatly expands the area of the grain boundary, complicates the path of crack growth, and also hinders the slip of the dislocation, resulting in a strengthening effect. On the other hand, the irregular geometry of these triangular protrusions prevents the relative sliding between grains at the grain boundaries, allowing the material to exhibit higher hardness.

#### 4.1.4. Internal Strengthening of Large Grains and the Uniform Stress Distribution Mechanism of a Dense Structure

The ratio of doping elements at the spinel tetrahedral interposition is 1:1:1:1 and the ionic radii vary little in Mg^2+^, Mn^2+^, Fe^2+^, and Zn^2+^, so widespread but slight lattice distortions may be induced in the overall crystal structure, as seen in the small changes in the cell parameters refined by XRD. This phenomenon may increase the local diffusion channel and thus increase the diffusion rate during grain growth.

Moreover, the uniform distribution of dopant elements effectively reduces the nucleation density during the early sintering stage. This occurs because the uniform distribution of dopants minimizes local compositional supersaturation, promoting a more diffuse and homogeneous nucleation process and preventing the formation of excessive small grains due to random nucleation. Simultaneously, the lattice distortion and local stress fields caused by doping increase the energy barrier for new crystal nucleation, suppressing excessive nucleation, and further reducing the nucleation density. The lower nucleation density provides sufficient diffusion resources for fewer grains, allowing them to grow steadily during sintering by consuming smaller grains through grain boundary migration. Prolonged high-temperature sintering further strengthens this process, ultimately yielding a dense microstructure with large grains.

The Hall–Petch effect improves hardness through grain boundary strengthening [53] as grain boundaries obstruct dislocation motion [54,55]. However, when the grain size exceeds the critical value, the grain boundary strengthening effect is weakened, the grain boundary sliding cannot become the main deformation mechanism, the deformation is transferred to the grain interior, and the opposite Hall–Petch effect may occur, and the hardness increases with the increase in the grain size. In this case, the increase in hardness depends more on the crystal structure itself, the distribution of defects within the grain, and the mechanisms that hinder the dislocation movement, such as dislocation entanglement and lattice distortion [56].

In addition, the dense and tightly packed large grains increase the grain boundary contact area so that the external load is more evenly distributed in the grain boundary. Friction and support between large grains further increase the hardness of the material. On the other hand, the high-hardness samples showed an apparent porosity rate of 1.35 ± 0.07%. So, the dense, pore-free structure significantly optimized the internal stress distribution of the material. The reduction in porosity leads to fewer stress concentrators, resulting in a more uniform overall stress distribution, which contributes to the improvement in hardness [57].

### 4.2. Mechanism of Flexural Strength Improvement in Samples (Al_2_Cr_0.5_Zn_0.1_Mg_0.2_Mn_0.2_)O_4_

**Figure 12:**
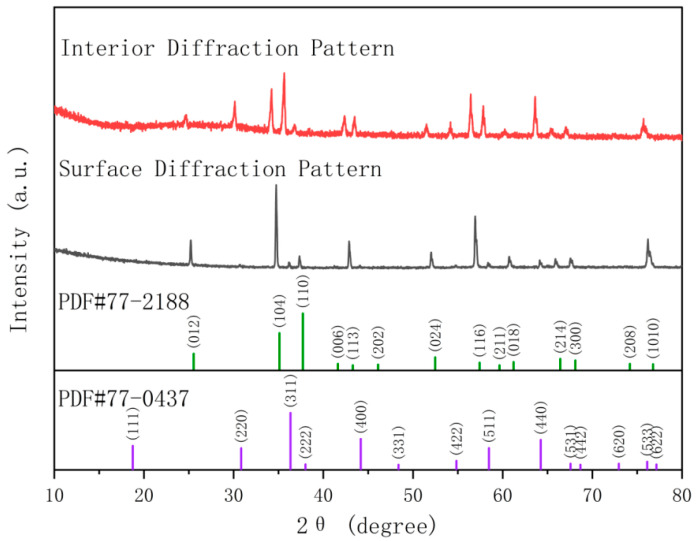
XRD patterns of the surface and interior of the high-flexural-strength sample.

In the high-flexural-strength sample, two phases are identified: the corundum-structured Al-Cr solid solution phase (Al_x_Cr_2−x_)O_3_ and the multi-element doped spinel phase (Zn_x_Mg_y_Mn_1−x−y_)(Al_a_Cr_1−a_)_2_O_4_. The comparative XRD patterns of the two phases are shown in Figure 12.

**Figure 13:**
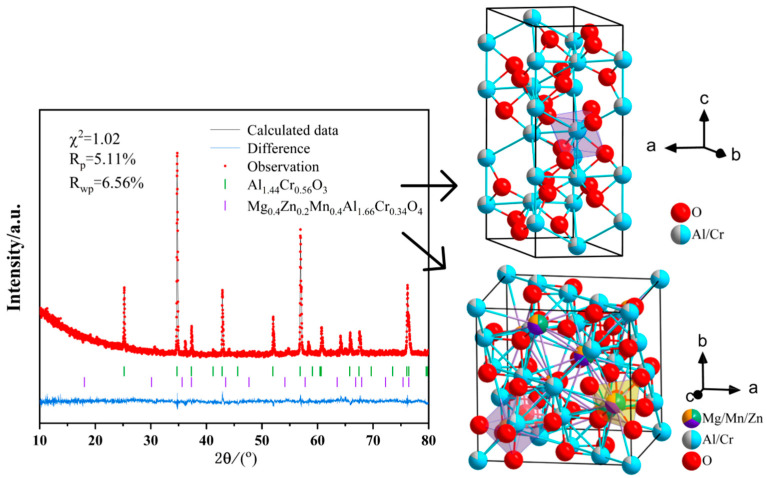
Rietveld-refined XRD patterns and schematic diagram of the crystal structure of the high-flexural-strength sample surface.

**Figure 14:**
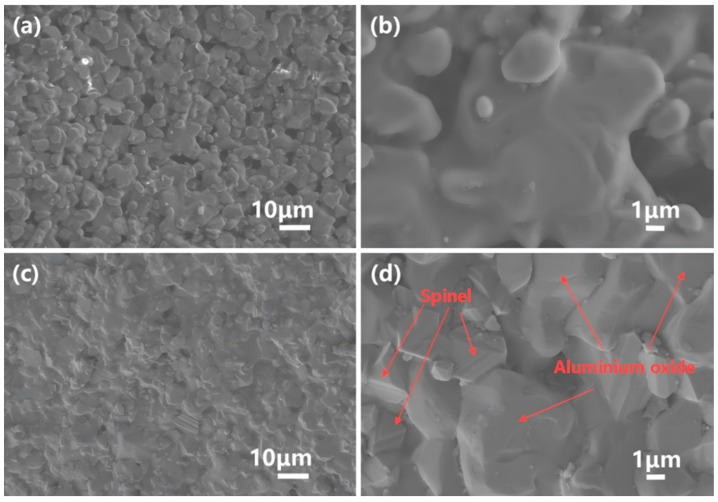
SEM images of the high-flexural-strength sample surface (**a**,**b**) and at a depth of 1 mm (**c**,**d**).

The XRD Rietveld refinement data for the sample surface, as shown in Figure 13 and Table A3, indicate that the surface is primarily composed of the corundum-structured Al-Cr solid solution phase, accounting for 94.3%, while the doped spinel phase accounts for 5.7%. The grain size on the sample surface ranges between 5 and 10 μm, with pore sizes around 2–5 μm. SEM observations, as shown in Figure 14a,b, reveal no detectable presence of the spinel phase, which is consistent with the XRD refinement results.

**Figure 15:**
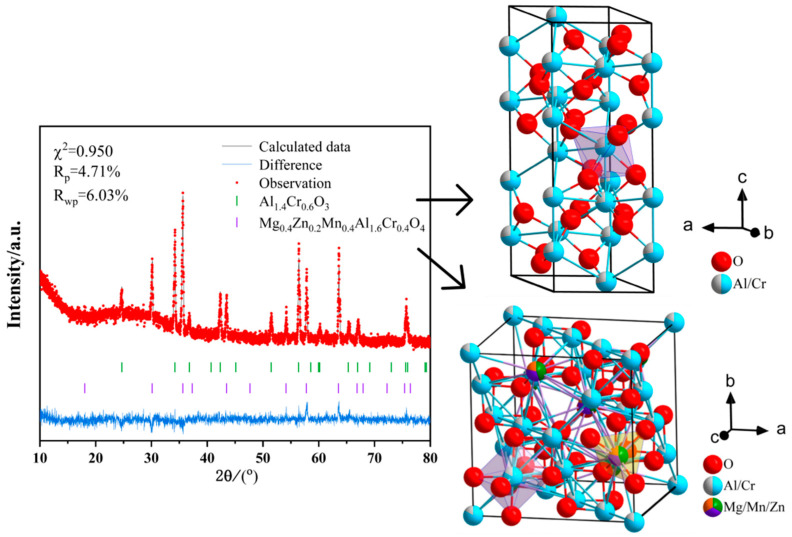
Rietveld-refined XRD patterns and schematic diagram of the crystal structure at a depth of 1 mm inside the high-flexural-strength sample.

**Figure 16:**
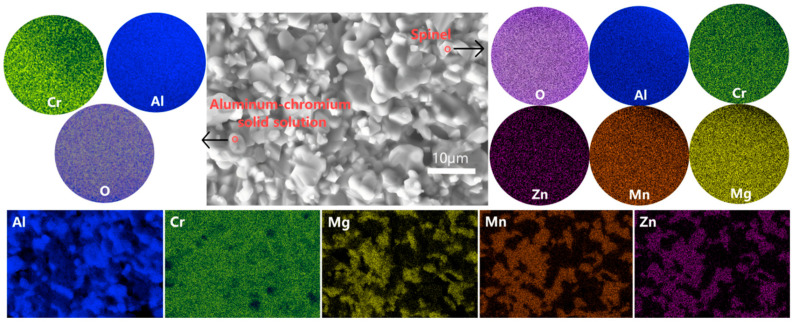
SEM and EDS images of the interior of the high-flexural-strength sample.

XRD Rietveld refinement data at a depth of 1mm from the sample surface are shown in Figure 15 and Table A4. Compared with the sample surface, the spinel phase proportion increases to 38.6%. SEM images (Figure 14c,d) display a dense, pore-free microstructure where the spinel phase and alumina phase coexist, exhibiting distinctly different morphologies. The spinel phase becomes significantly more abundant, which is consistent with the refinement data. In the circular area of Figure 16, the Al-Cr solid solution phase is observed to contain Al and Cr elements, while the spinel phase contains Al, Cr, Mg, Mn, and Zn elements. Notably, Al-Cr elements are present in both phases, with nearly identical Cr content. Consequently, the Cr distribution map in the lower part of Figure 16 shows no obvious phase boundary, as Cr appears to be uniformly distributed. In contrast, Mg, Mn, and Zn elements are exclusively present in the spinel phase, which can be directly observed. The elemental content aligns well with the XRD refinement data.

For materials with a corundum-structured Al-Cr solid solution phase on the surface and a mixed structure of doped spinel phase and Al-Cr solid solution phase in the interior, the impact on flexural strength can be attributed to solid solution strengthening, second-phase strengthening, a low porosity structure, and a phase concentration gradient from the surface to the interior.

#### 4.2.1. Solid Solution Strengthening in Both Phases

Due to the ionic radius differences in the solute ions occupying different interstitial positions, solid solution strengthening occurs in both phases [58,59]. The extensive lattice distortions induced by solid solution formation require dislocations to overcome higher energy barriers during slip. Additionally, the interaction between solute atoms and dislocations generates local stress fields or potential wells, which act as “pinning” centers for dislocations. This pinning effect further enhances resistance to dislocation motion, improving the material’s flexural strength.

#### 4.2.2. Low-Porosity Structure

The surface layer is primarily dominated by an Al-Cr solid solution with a corundum structure. The addition of the chromium solid solution improves the sintering performance and reduces the number of pores. The material interior, compared to the surface layer, is more compact, exhibiting fewer pores under SEM observation (Figure 14c,d). The show porosity of the sample with high flexural strength is 2.35 ± 0.11%. The reduction in porosity leads to a more compact structure, reducing the propagation paths of cracks and defects. Porosity is a weak point in ceramic materials, often acting as a stress concentrator, which can result in fracture or failure under external forces. As porosity decreases, the material’s density increases, allowing for more efficient distribution of applied stress, thus improving its flexural strength [60].

#### 4.2.3. The Dense Distribution of the Internal Spinel Phase and Al-Cr Solid Solution Phase

The dense and uniform distribution of the spinel and Al-Cr solid solution phases, without phase agglomeration or segregation (as shown in Figure 16), ensures that the spinel phase contributes significantly to second-phase strengthening. The finely distributed second-phase particles enhance the material’s mechanical properties primarily through particle strengthening and interface strengthening mechanisms. The second-phase particles act as obstacles to dislocation motion, a mechanism known as particle strengthening, which occurs when dislocations encounter these particles and must either bypass them or accumulate stress around them. This resistance to dislocation motion increases the material’s flexural strength, making it more resistant to bending and fracture under stress. The uniform distribution of these second-phase particles ensures that the reinforcing effect is consistent across the material, providing effective resistance to dislocation movement throughout the entire structure.

In addition to particle strengthening, the interface between the second-phase particles and the matrix also plays a significant role in enhancing the material’s strength. The grain boundaries or phase interfaces serve as barriers to dislocation slip and grain boundary sliding as dislocations encounter the lattice mismatch and stress concentration at the interface. The denser and more uniform the distribution of the second-phase particles, the more effective these interfaces are at hindering dislocation movement, further improving the material’s deformation resistance. This combination of particle and interface strengthening significantly enhances the material’s overall resistance to deformation, which directly contributes to the increase in flexural strength.

#### 4.2.4. Phase Concentration Gradient from the Surface to the Interior

The phase concentration gradient from the sample surface to the interior induces a transition in the material’s microstructure, leading to distinct differences in lattice structure, elastic modulus, and slip systems between the phases. These differences create varied crystal environments, which affect the behavior of cracks or dislocations as they propagate inward from the surface.

As microcracks or dislocations extend inward, they first propagate through a polycrystalline environment dominated by the corundum phase. However, as the concentration of the spinel phase increases deeper into the material, the microstructure transitions to a more uniform dual-phase polycrystalline structure. This shift in the crystal environment creates resistance to crack propagation as the cracks or dislocations must navigate through different phase boundaries, each with varying mechanical properties.

The phase concentration gradient imposes additional energy barriers to the movement of cracks and dislocations, effectively “pinning” them at the phase boundaries and reducing the likelihood of crack growth. This gradient-controlled transition in the crystal structure enhances the material’s flexural strength by hindering the advancement of cracks and dislocations, resulting in a more robust and durable material under bending stress.

## 5. Conclusions

In this study, we successfully developed two types of multi-element spinel refractories, sintered at 1600 °C for two hours. The (Al_2_Fe_0.25_Zn_0.25_Mg_0.25_Mn_0.25_)O_4_ refractory demonstrated a Vickers hardness of 1770.6 ± 79.1 HV1, which is significantly higher than MgAl_2_O_4_ and MgCr_2_O_4_, while the (Al_2_Cr_0.5_Zn_0.1_Mg_0.2_Mn_0.2_)O_4_ refractory exhibited an excellent flexural strength of 161.2 ± 9.7 MPa. These improvements are attributed to mechanisms such as solid solution strengthening, second-phase strengthening, and phase concentration gradient distribution. Additionally, the successful application of machine learning models, specifically Random Forest and Neural Networks, enabled the high-throughput prediction of room temperature flexural strength and microhardness, demonstrating the potential of machine learning for optimizing material performance.

Key findings of this research include the following:

1. The successful development of multi-element spinel refractories with significantly enhanced hardness and flexural strength.
2. Identification of the key strengthening mechanisms, including solid solution strengthening, second-phase strengthening, and phase concentration gradients.
3. The effective use of machine learning algorithms for high-throughput material property prediction, enabling the precise optimization of the refractories’ performance.

In summary, the introduction of elements such as aluminum, chromium, iron, zinc, magnesium, and manganese has endowed spinel refractories with an outstanding performance. The differences in the types and contents of these elements further contribute to the material’s exceptional properties in various aspects. This highlights the great potential of multi-element complex composition optimization in high-temperature applications and offers a new perspective for optimizing its performance. In addition, the successful application of machine learning methods and models in performance prediction highlights their effectiveness as a powerful tool to accelerate the discovery and development of advanced materials, providing new technical means for refractories research.

Looking ahead, future research will focus on two key aspects:

1. Expanding the dataset for more accurate prediction models: By increasing the volume and diversity of the data used for training machine learning models, we aim to enhance the accuracy and reliability of predictions related to refractory performance. This will ensure the better optimization and customization of material properties for specific applications.
2. Customizing refractory materials to meet industry-specific needs: The next step will be to integrate the diverse and evolving demands of the refractory industry into the development process, creating functionalized, tailor-made refractories for specific application scenarios. By focusing on additional performance indicators, such as high-temperature wear resistance and thermal shock resistance, we aim to provide targeted solutions that better address the complex requirements of refractory materials in different industrial sectors.

## Figures and Tables

**Table 1:** The 1200 °C sintering excellent mechanical properties formula comparison table.

Formula	Flexural Strength (MPa)	Microhardness (HV1)
(Al_2_Fe_0.5_Zn_0.1_Mg_0.2_Mn_0.2_)O_4_	82.9 ± 4.1	196.7 ± 10.9
(Al_0.67_Cr_0.67_Fe_0.66_Zn_0.5_Cu_0.5_)O_4_	50.0 ± 2.7	1112.6 ± 44.9
MgAl_2_O_4_	6.3 ± 1.9	22.8 ± 3.6
MgCr_2_O_4_	14.7 ± 0.47	139.9 ± 8.5

Values represent mean ± standard deviation.

**Table 2:** The 1400 °C sintering excellent mechanical properties formula comparison table.

Formula	Flexural Strength (MPa)	Microhardness (HV1)
(Al_2_Fe_0.5_Zn_0.1_Mg_0.2_Mn_0.2_)O_4_	148.4 ± 8.7	791.1 ± 36.3
(Al_0.67_Fe_0.66_Cr_0.67_Zn_0.4_Mg_0.4_Cu_0.2_)O_4_	58.1 ± 3.9	863.4 ± 25.4
(Al_0.67_Fe_0.66_Cr_0.67_Zn_0.33_Mg_0.33_Cu_0.34_)O_4_	132.0 ± 5.9	924.2 ± 36.9
MgAl_2_O_4_	14.3 ± 2.8	58.1 ± 5.2
MgCr_2_O_4_	21.9 ± 2.9	122.4 ± 5.0

Values represent mean ± standard deviation.

**Table 3:** The 1600 °C sintering excellent mechanical properties formula comparison table.

Formula	Flexural Strength (MPa)	Microhardness (HV1)
(Al_2_Cr_0.5_Zn_0.1_Mg_0.3_Mn_0.1_)O_4_	130.3 ± 8.1	567.7 ± 19.3
(Al_2_Fe_0.25_Zn_0.35_Mg_0.15_Mn_0.25_)O_4_	70.6 ± 6.0	1547.1 ± 80.2
MgAl_2_O_4_	114.7 ± 4.7	528.4 ± 17.3
MgCr_2_O_4_	39.2 ± 3.2	105.6 ± 4.7

Values represent mean ± standard deviation.

**Table 4:** The 24 descriptors whose absolute value of the Pearson coefficient is less than 0.9.

Weighted Average	Weighted Standard Deviation
1. Relative atomic weight 2. Melting point 3. Boiling point 4. Electronegativity 5. Number of valence electrons 6. Lattice constant a 7. Specific heat capacity 8. Thermal conductivity coefficient 9. Melting point of oxides 10. Oxide lattice constant a 11. Oxide lattice constant c 12. Configurational entropy 13. Firing temperature of the sample (T)	14. SD_Relative atomic weight 15. SD_Melting point 16. SD_Boiling point 17. SD_Covalent radius 18. SD_First ionization energy 19. SD_Electronegativity 20. SD_Number of valence electrons 21. SD_Atomic radius 22. SD_Thermal conductivity coefficient 23. SD_Oxides molecular weight 24. SD_Melting point of oxides

**Table 5:** R^2^ value of flexural strength training evaluation of 8 algorithms.

	Algorithm	ADA	DTR	EN	LASSO	NN	RF	RIDGE	XGB
Number of Descriptors									
4		0.566	0.263	0.413	0.413	−3.090	0.560	0.185	0.451
8		0.563	0.295	0.391	0.390	0.471	0.571	0.216	0.410
12		0.570	0.366	0.392	0.391	0.532	0.568	0.321	0.404
16		0.550	0.298	0.392	0.391	0.431	0.567	0.327	0.414
20		0.566	0.318	0.395	0.394	0.533	0.555	0.327	0.401
24		0.501	0.311	0.395	0.394	0.460	0.557	0.327	0.395

**Table 6:** R^2^ value of microhardness training evaluation of 8 algorithms.

	Algorithm	ADA	DTR	EN	LASSO	NN	RF	RIDGE	XGB
Number of Descriptors									
4		0.440	0.193	0.362	0.361	−0.282	0.322	0.050	0.519
8		0.444	0.293	0.348	0.346	0.574	0.541	0.052	0.503
12		0.422	0.147	0.374	0.367	−0.082	0.549	0.285	0.489
16		0.390	0.181	0.374	0.367	0.571	0.532	0.284	0.433
20		0.356	0.184	0.378	0.367	0.514	0.515	0.266	0.453
24		0.391	0.222	0.356	0.350	0.561	0.509	0.266	0.482

**Table 7:** Algorithm hyperparameter selection.

Random Forest Hyperparameters	Neural Network Hyperparameters
n_estimators = 54 (number of trees in the forest)	hidden_layer_sizes = (148,148) (number of neurons in each hidden layer)
max_depth = 8 (maximum depth of each tree)	activation = ‘relu’ (activation function for hidden layers)
min_samples_split = 2 (minimum number of samples required to split an internal node)	alpha = 0.061 (L2 regularization term)
min_samples_leaf = 1 (minimum number of samples required to be at a leaf node)	learning_rate = ‘constant’ (learning rate schedule)
max_features = ‘log2’ (number of features to consider when looking for the best split)	learning_rate_init = 0.001 (initial learning rate)
criterion = ‘friedman_mse’ (function to measure the quality of a split)	max_iter = 1026 (maximum number of iterations)

## Data Availability

The original contributions presented in this study are included in the article. Further inquiries can be directed to the corresponding author.

## Abbreviations

The following abbreviations are used in this manuscript:

ADA	Adaptive Boosting
CCCs	Compositionally complex ceramics
DAGAN	Data augmentation generative adversarial network
DFT	Density functional theory
DTR	Decision Tree Regression
EDS	Energy Dispersive Spectroscopy
EN	Elastic Net
HECs	High-entropy ceramics
HEOs	High-entropy oxides
HENs	High-entropy nitrides
HV1	Vickers hardness (1 kgf load)
ISO	International Organization for Standardization
KNN	K-nearest neighbors
LASSO	Least Absolute Shrinkage and Selection Operator
ML	Machine learning
MPCCs	Multi-principal cation ceramics
NN	Neural Network
RF	Random Forest
R^2^	Coefficient of Determination
Ridge	Ridge Regression
RMSE	Root Mean Square Error
SEM	Scanning Electron Microscopy
SHAP	Shapley Additive Explanations
XGB	Extreme Gradient Boosting
XRD	X-ray diffraction

## Appendix B

**Table A1:** All 48 descriptors.

The Weighted Average of the Properties of Elements	Element Property Weighted Standard Deviation	The Weighted Average of Oxide Properties	Oxide Properties Weighted Standard Deviation	Other Descriptors
Relative atomic weight	SD_Relative atomic weight	Oxide molecular weight	SD_Oxides molecular weight	Mixed entropy
Density	SD_Density	Melting point of oxides	SD_Melting point of oxides	Firing T
Melting point	SD_Melting point	Oxide density	SD_Oxide density	
Boiling point	SD_Boiling point	Oxide lattice constant a	SD_Oxide lattice constant a	
Covalent radius	SD_Covalent radius	Oxide lattice constant c	SD_Oxide lattice constant c	
First ionization energy	SD_First ionization energy	Standard Enthalpy of Formation	SD_Standard Enthalpy of Formation	
Electronegativity	SD_Electronegativity	Standard Molar Entropy	SD_Standard Molar Entropy	
Number of valence electrons	SD_Number of valence electrons	Standard Gibbs free energy	SD_Standard Gibbs free energy	
Lattice constant a	SD_Lattice constant a	Predicted formation energy	SD_Predicted formation energy	
Atomic radius	SD_Atomic radius			
Melting enthalpy	SD_Melting enthalpy			
Enthalpy of vaporization	SD_Enthalpy of vaporization			
Specific heat capacity	SD_Specific heat capacity			
Thermal conductivity coefficient	SD_Thermal conductivity coefficient			

The raw XRD data used for the refinement were collected from flat bulk samples over a 2θ range of 10–80°, with a step size of 0.013° and a step time of 10.200 s.

**Table A2:** Rietveld-Refined XRD data table of the high-hardness sample.

Cell Parameters	a (Å)	b (Å)	c (Å)	Alpha (°)	Volume (Å^3^)
(Al_2_Fe_0.25_Zn_0.25_Mg_0.25_Mn_0.25_)O_4_	8.08169	8.08169	8.08169	90	527.8459
Atomic coordinates and occupancy rates	X	Y	Z	Occupancy	B isotropic
O1	0.252776	0.252776	0.252776	1	0.615073
Al1	0.5	0.5	0.5	1	0.36478
Fe1	0.125	0.125	0.125	0.245584	0.355306
Zn1	0.125	0.125	0.125	0.240822	0.355306
Mg1	0.125	0.125	0.125	0.266758	0.355306
Mn1	0.125	0.125	0.125	0.246837	0.355306

**Table A3:** Rietveld-Refined XRD data table for the surface of the high-flexural-strength sample.

Cell Parameters	a (Å)	b (Å)	c (Å)	Alpha (°)	Volume (Å^3^)
(Al_1.66_Cr_0.34_Zn_0.2_Mg_0.4_Mn_0.4_)O_4_	8.18423	8.18423	8.18423	90/90/90	548.193
Al_1.44_Cr_0.56_O_3_	4.78885	4.78885	13.06658	90/90/120	259.5105
(Al_1.66_Cr_0.34_Zn_0.2_Mg_0.4_Mn_0.4_)O_4_ Atomic coordinates and occupancy rates	X	Y	Z	Occupancy	B isotropic
O1	0.235234	0.235234	0.235234	1	0.5
Al1	0	0	0	0.830134	0.2
Cr1	0	0	0	0.169866	0.2
Zn1	0.375	0.375	0.375	0.200602	0.3
Mg1	0.375	0.375	0.375	0.400982	0.3
Mn1	0.375	0.375	0.375	0.398416	0.3
Al_1.44_Cr_0.56_O_3_ Atomic coordinates and occupancy rates	X	Y	Z	Occupancy	B isotropic
O1	0.317371	0	0.25	1	0.13
Al1	0	0	0.148512	0.721197	0.25
Cr1	0	0	0.148512	0.278803	0.25

**Table A4:** Rietveld-Refined XRD data table for the interior of the high-flexural-strength sample.

Cell Parameters	a (Å)	b (Å)	c (Å)	Alpha (°)	Volume (Å^3^)
(Al_1.6_Cr_0.4_Zn_0.2_Mg_0.4_Mn_0.4_)O_4_	8.19618	8.19618	8.19618	90/90/90	550.5986
Al_1.4_Cr_0.6_O_3_	4.79338	4.79338	13.07707	90/90/120	260.2102
(Al_1.6_Cr_0.4_Zn_0.2_Mg_0.4_Mn_0.4_)O_4_ Atomic coordinates and occupancy rates	X	Y	Z	Occupancy	B isotropic
O1	0.2395	0.2395	0.2395	1	0.5
Al1	0	0	0	0.830727	0.2
Cr1	0	0	0	0.169273	0.2
Zn1	0.375	0.375	0.375	0.215831	0.3
Mg1	0.375	0.375	0.375	0.443262	0.3
Mn1	0.375	0.375	0.375	0.340907	0.3
Al_1.4_Cr_0.6_O_3_ Atomic coordinates and occupancy rates	X	Y	Z	Occupancy	B isotropic
O1	0.304534	0	0.25	1	0
Al1	0	0	0.147805	0.72151	0.98
Cr1	0	0	0.147805	0.27849	0.98
